# Small animal PET imaging with the ^68^Ga-labeled pH (low) insertion peptide-like peptide YJL-4 in a triple-negative breast cancer mouse model

**DOI:** 10.1186/s41181-024-00267-x

**Published:** 2024-04-27

**Authors:** YueHua Chen, ShuangShuang Song, YanQin Sun, FengYu Wu, GuangJie Yang, ZhenGuang Wang, MingMing Yu

**Affiliations:** Department of Nuclear Medicine, The Affiliated Hospital of Qingdao University, Qingdao, 266100 China

**Keywords:** Triple-negative breast cancer, Tumor microenvironment, pH (low) insertion peptides, ^68^Ga, Small animal PET/CT, Molecular imaging

## Abstract

**Background:**

The aim of this study was to prepare a novel ^68^Ga-labeled pH (low) insertion peptide (pHLIP)-like peptide, YJL-4, and determine its value for the early diagnosis of triple-negative breast cancer (TNBC) via in vivo imaging of tumor-bearing nude mice. The novel peptide YJL-4 was designed using a template-assisted method and synthesized by solid-phase peptide synthesis. After modification with the chelator 1,4,7‑triazacyclononane-N,N′,N″-triacetic acid (NOTA), the peptide was labeled with ^68^Ga. Then, the biodistribution of ^68^Ga-YJL-4 in tumor-bearing nude mice was investigated, and the mice were imaged by small animal positron emission tomography (PET).

**Results:**

The radiochemical yield and radiochemical purity of ^68^Ga-YJL-4 were 89.5 ± 0.16% and 97.95 ± 0.06%, respectively. The biodistribution of ^68^Ga-YJL-4 in tumors (5.94 ± 1.27% ID/g, 6.72 ± 1.69% ID/g and 4.54 ± 0.58% ID/g at 1, 2 and 4 h after injection, respectively) was significantly greater than that of the control peptide in tumors at the corresponding time points (*P* < 0.01). Of the measured off-target organs, ^68^Ga-YJL-4 was highly distributed in the liver and blood. The small animal PET imaging results were consistent with the biodistribution results. The tumors were visualized by PET at 2 and 4 h after the injection of ^68^Ga-YJL-4. No tumors were observed in the control group.

**Conclusions:**

The novel pHLIP family peptide YJL-4 can adopt an *α-*helical structure for easy insertion into the cell membrane in an acidic environment. ^68^Ga-YJL-4 was produced in high radiochemical yield with good stability and can target TNBC tissue. Moreover, the strong concentration of radioactive ^68^Ga-YJL-4 in the abdomen does not hinder the imaging of early TNBC.

**Supplementary Information:**

The online version contains supplementary material available at 10.1186/s41181-024-00267-x.

## Background

Breast cancer is the most common cancer in women, accounting for 31% of female cancer cases, and the incidence rate of breast cancer is increasing by approximately 0.5% per year (Siegel et al. 2023). Triple-negative breast cancer (TNBC) is a high-risk subtype of breast cancer that is prone to metastasis and has high recurrence and a poor prognosis. Imaging methods for the early diagnosis of breast cancer mainly include mammography, magnetic resonance imaging (MRI) and ultrasound (Senkus et al. 2015). However, TNBC commonly occurs in young women (Jitariu et al. 2017), and the breast tissue of this population is relatively dense, which reduces the sensitivity of mammography and ultrasound (Ditsch et al. 2021). Although MRI has a relatively high sensitivity, the false positive rate is also high (Ditsch et al. 2021; Bakker et al. 2019). Fluorine-18 fluorodeoxyglucose positron emission tomography/computed tomography (^18^F-FDG-PET/CT) has also been used in the diagnosis and staging of TNBC (Ulaner et al. 2016; Yue et al. 2015). However, for mastitis, this imaging technique may lead to false-positive results (de Jong et al. 2023). Therefore, a method for the effective early diagnosis and screening of TNBC is urgently needed.

The tumor microenvironment (TME) of almost all solid tumors is acidic, with a pH ranging between 6.0 and 6.5 (de la Cruz-López et al. 2019). The TME is stable and not affected by the clonal evolution of tumors. Therefore, targeting the TME has more advantages than targeting tumor cell membrane receptors. pH (low) insertion peptides (pHLIPs) are sensitive to changes in pH; the acidic amino acid residues in pHLIPs are protonated in the acidic TME, forming an α-helix structure that can be inserted into the tumor cell membrane (Gupta and Mertz 2017; Weerakkody et al. 2013; Pathak et al. 2004; Penet et al. 2008; Wyatt et al. 2018). Therefore, pHLIPs are expected to be novel tumor-targeting carriers suitable not only for imaging TNBC but also for transporting therapeutic molecules to TNBC. PHLIPs are short peptides containing approximately 30 amino acids that can target the TME and can insert therapeutic molecules (including polar molecules that are difficult to transport through the cell membrane) with their C-termini linked into tumor cells. In comparison to other targeted TNBC drug delivery vehicles, including specialized nanosized delivery vehicles (Greish et al. 2018; Krausz et al. 2018; Sorolla et al. 2019; Valcourt et al. 2019) and macrophage-derived extracellular vesicles (Haney et al. 2020), the synthesis of this drug delivery vehicle is straightforward and inexpensive, and it can more easily transport therapeutic molecules into tumor cells. Our group’s previous studies used pHLIPs as carriers to target MDA-MB-231 TNBC cells (Chen et al. 2021, 2022; Wu et al. 2022; Yu et al. 2020, 2021). These studies demonstrated that although pHLIPs could target tumor cells, the binding of the probe to the tumor was not sufficiently strong. To improve the targeting of the probe, we compared the amino acid sequences of the pHLIPs and found significant positional conservation of the amino acids in the α-helix regions of the pHLIPs. Next, we used the template-assisted method (Tossi et al. 2000) to select the amino acid with the highest frequency at each position in the α-helix regions of pHLIPs and produce a new pHLIP‑like peptide, YJL-4. This study aimed to investigate the diagnostic efficacy of positron emission tomography/computed tomography (PET/CT) for TNBC using ^68^Ga-labeled YJL-4 (^68^Ga-YJL-4).

## Methods

### Main instruments

The main equipment used in this study included a Chirascan plus ACD spectropolarimeter (Applied Photophysics, United Kingdom), a CRC-55tR radiopharmaceutical dose calibrator (Capintec Inc., USA), a Wizard 1480 gamma counter (PerkinElmer Instruments Inc., USA), a 20-mCi ^68^Ge/^68^Ga generator (Obninsk Cyclotron Co., Ltd., Russia), and an Inveon small animal PET scanner (Siemens, Germany).

### Main reagents

The main reagents used in this study included isoflurane (Shenzhen RWD Life Technology Co., Ltd., China) and hydrochloric acid (Nanjing Chemical Reagent Co., Ltd., China).

### Cell line and experimental animals

Human breast cancer cells (MDA-MB-231 cells from the Cell Bank of the Chinese Academy of Sciences) were cultured in high-glucose DMEM supplemented with 10% fetal bovine serum and 1% double antibiotic mixture (penicillin‒streptomycin mixture) at 37 °C in 5% CO_2_. Twenty-four four- to five-week-old female specific pathogen-free (SPF) BALB/c nude mice (Hangzhou Ziyuan Experimental Animal Technology Co., Ltd., China, license number: SCXK(Zhe) 2019–0004) were used to establish the MDA-MB-231 tumor model by subcutaneously inoculating 0.1 mL of cell suspension (50 µL of PBS + 50 µL of Matrigel) containing 1 × 10^6^ MDA-MB-231 cells at the base of the right forelimb of each mouse. The animal studies were approved by the ethics committee of Qingdao University, and the management of the animals adhered to the animal ethics standards.

### Design and characterization of YJL-4

**Fig. 1:**
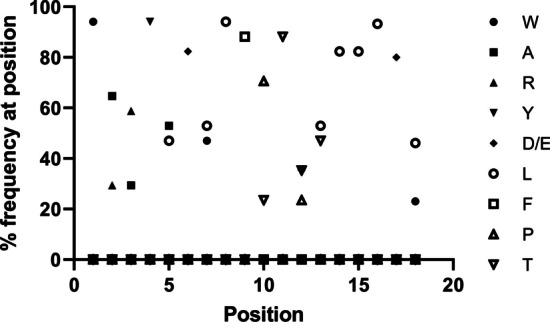
Distribution of amino acids at different positions in the insertion regions of pHLIPs

The amino acids in the insertion regions of pHLIPs that can form α-helices were analyzed, and the amino acids at each position were conserved to a certain extent. The dominant amino acids in the insertion regions of the pHLIPs were selected to obtain a new peptide, YJL-4 (Fig. 1).

YJL-4: ACDDQNPWARYADLLFPTDTLLLDL-NH_2_

The ProtParam (https://web.ExPASy.org/protparam/) and HeliQuest (http://heliquest.ipmc.cnrs.fr/) online tools were used to analyze the instability index, net charge, grand average of hydropathicity (GRAVY), hydrophobicity and hydrophobic moment of the cell membrane insertion region of the peptides. PyMOL, HeliQuest, ProtScale and DNASTAR were used to analyze and predict the secondary structure of the new peptide.

### Synthesis and purification of peptides

**Fig. 2:**
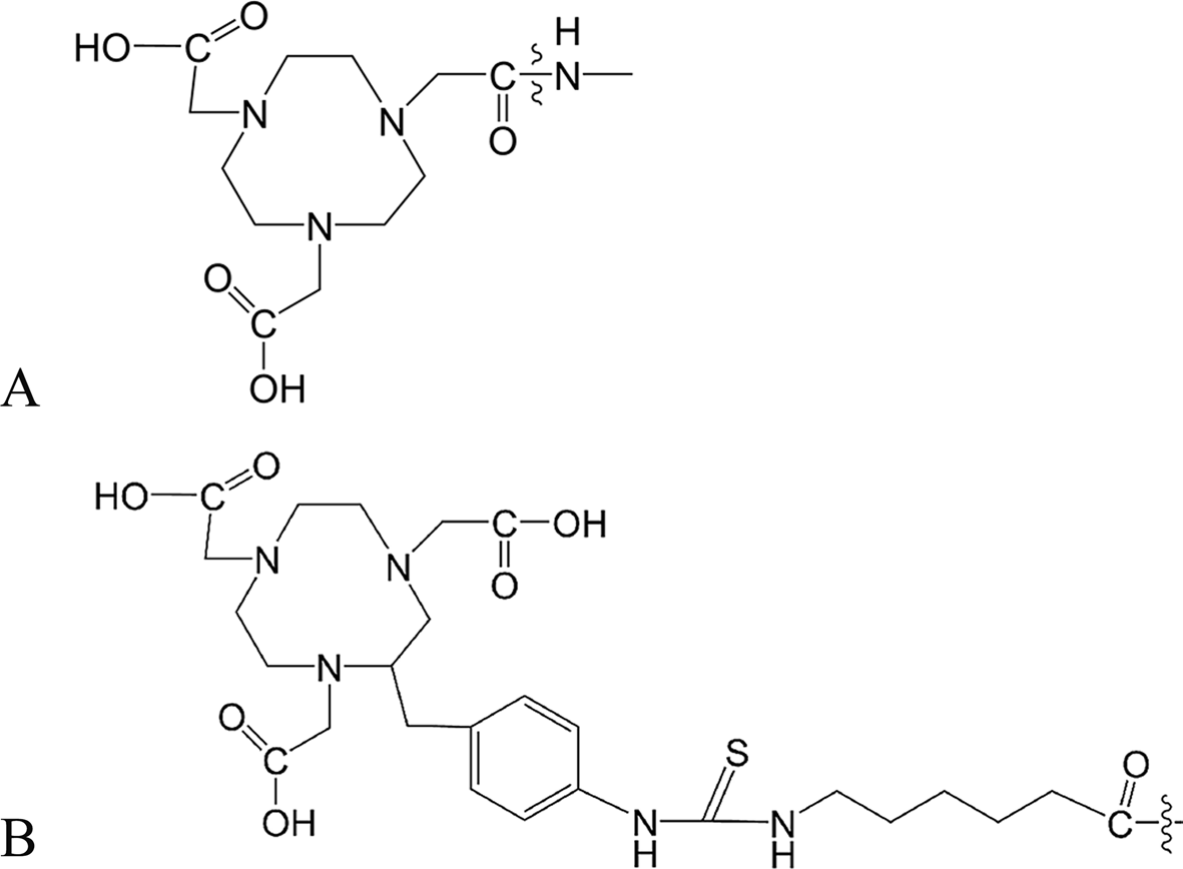
NOTA chelator used in previous studies (**A**) and this study (**B**)

YJL-4 and the control peptide, kVar7 (Sosunov et al. 2013), were synthesized via solid-phase peptide synthesis, and the amino group of the N-terminal amino acid of the peptide was connected to the 1,4,7‑triazacyclononane-N,N′,N″-triacetic acid (NOTA) chelator (Fig. 2B) through a condensation reaction. NOTA-YJL-4 and NOTA-kVar7 were purchased from Shanghai Science Peptide Biological Technology Co., Ltd. The peptide sequences used were as follows:

YJL-4: NOTA-ACDDQNPWARYADLLFPTDTLLLDL-NH_2_
kVar7: NOTA-ACEEQNPWARYLKWLFPTKTLLLKL-NH_2_

YJL-4 and kVar7 were purified using a Shimadzu LC-20AT high-performance liquid chromatography (HPLC) system (Shimadzu Corporation, Japan) with the following settings: column: Agela C18 column (250 mm × 4.6 mm, 5 μm); mobile phase: solvent A (0.05% trifluoroacetic acid + 2% acetonitrile), solvent B (0.05% trifluoroacetic acid + 90% acetonitrile); gradient elution (0–20 min, 40–60% B); flow rate: 1.0 mL/min; and detection wavelength: 220 nm. The peptides were identified using a Shimadzu LCMS-2020 system (Shimadzu Corporation, Japan) to determine the molecular characteristics of the synthesized peptide molecules.

### Circular dichroism (CD) spectroscopy

The peptide (0.1 mg/mL) and 1-palmitoyl-2-oleoyl-*sn*-glycero-3-phosphocholine (POPC, 2 mmol/L, diameter ≤ 50 nm) were dissolved in phosphate buffer (PB, 10 mmol/L, pH = 8) and then equilibrated at room temperature for at least 10 h. The pH of the solution was adjusted to 4.0 using 0.1 mol/L HCl.

CD spectra of the peptide were measured with a Chirascan plus ACD spectropolarimeter at pH = 8.0 and 4.0 with the following settings: optical path of the quartz sample cell, 1 mm; scanning wavelength range, 190–260 nm; wavelength step size, 1 nm; time-per-point, 0.5 s; and temperature, 25 °C.

### Preparation and purification of ^68^Ga-labeled peptides

After the ^68^Ge/^68^Ga generator was rinsed with 5 mL of 0.1 M HCl, the eluate was collected fractionally, and the fraction with the highest radioactivity was used for subsequent labeling. A 1.5-mL centrifuge tube was used as the reaction tube. First, 500 μL of 0.1 M sodium acetate buffer (pH = 4.5), 190.55 MBq/500 μL of ^68^Ga eluent, and 80 μL of 0.1 mg/mL NOTA**-**YJL-4 (or kVar7) were sequentially added to the tube and then mixed by vortexing for 30 s at 800 rpm and heated at 40 °C for 15 min. After decreasing to room temperature, the solution was added to the activated Waters Sep-Pak C-18 column, and the column was then rinsed with 3 mL of ultrapure water and finally with 1.0 mL of 80% ethanol to obtain the purified labeled product.

The ^68^Ga-labeled peptides were identified using an Agilent 1200 radio-HPLC instrument (Agilent Technologies Inc., USA) under the following settings: mobile phase A: 0.1% trifluoroacetic acid aqueous solution; mobile phase B: acetonitrile; elution gradient: 0–15 min, 95–5% mobile phase A and 5–95% mobile phase B; and flow rate: 1 mL/min. The radioactive peak retention time was observed.

### Serum stability and serum protein binding

Two hundred microliters of each ^68^Ga-labeled peptide (18.5 MBq) was mixed with 1.0 mL of fresh human serum (or mouse serum) and incubated at 37 °C, and the radiochemical purity was detected at 30, 60, 120, and 240 min after mixing.

The radiotracer (0.37 MBq, 100 µl) was incubated while stirring (600 rpm) in mouse serum at 37 °C. At 30, 60, 120, and 240 min, 250 µl of serum was transferred to a 1.7 ml Eppendorf tube containing ice-cold acetonitrile (250 µl). The resulting mixture was vortexed for 1 min and centrifuged (13,000 rpm) for 5 min. The supernatant was transferred to a 1.7 ml Eppendorf tube and then further centrifuged at 13,000 rpm for 2 min. The supernatant and sediment were collected separately, and the radioactivity was measured with a gamma counter to determine the serum protein binding of the radiotracer.

### In vivo biodistribution

Eighteen tumor-bearing nude mice were selected for in vivo biodistribution experiments when the tumor diameter reached 0.8–1.0 cm. Mice were randomly divided into two groups: a YJL-4 group and a control group with 9 mice per group; each group had three time points. Each nude mouse was injected with 1.85 MBq/200 μL of ^68^Ga-YJL-4 or ^68^Ga-kVar7 through the tail vein. The mice were humanely sacrificed 1 h, 2 h, or 4 h after injection. The brain, heart, lungs, liver, kidneys, stomach, small intestine, blood, muscles, bones, and tumor were collected and weighed, and the radioactivity was measured. The biodistribution results are expressed as the percent of the injected dose per gram of tissue (% ID/g).

### Small animal PET imaging

Six tumor-bearing nude mice were selected for imaging when the tumor diameter reached 0.8–1.0 cm. Mice were randomly divided into two groups: a YJL-4 group and a control group, with 3 mice per group; each group had two time points. Each tumor-bearing nude mouse was injected with approximately 7.4 MBq/200 μL of ^68^Ga-YJL-4 or ^68^Ga-kVar7 through the tail vein, and the mice were anesthetized with 2% isoflurane at 2 and 4 h after injection and imaged using static 10-min PET and moderate-resolution whole-body CT. Data processing was performed using the Innovon Research Workstation, and images were reconstructed by ordered subset expectation maximization (OSEM).

### Statistical analysis

SPSS 24.0.0.0 statistical software (IBM) was used for data processing. Quantitative data are expressed as the mean (M) ± standard deviation (SD). Variables were compared using one-way analysis of variance (ANOVA), and the homogeneity of variance was tested using Levene's test. A *P* value < 0.05 was considered to indicate statistical significance.

## Results

### Bioinformatics analysis of the novel peptide

**Fig. 3:**
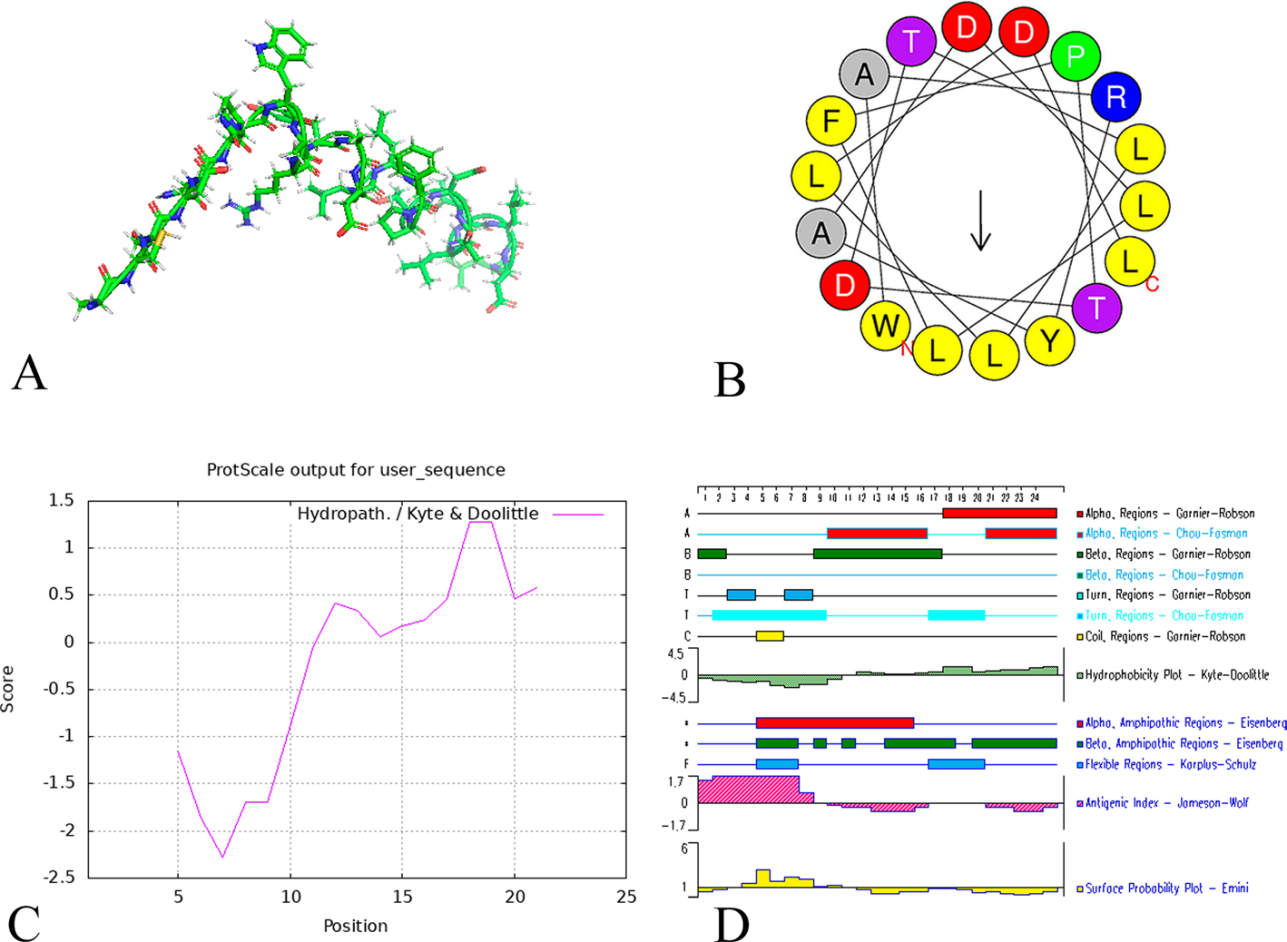
A. The molecular structure of YJL-4. B. Helical wheel projections showing the exact spatial positions and arrangement of polar and nonpolar amino acids in the YJL-4 insertion region. C. ProtScale tool analysis showing that the C-terminus of the peptide is hydrophobic, while the N-terminus is hydrophilic. D. DNASTAR analysis indicating that YJL-4 has an α-helical structure and is amphiphilic

(1) Analysis of basic peptide properties: The basic parameters (instability index, net charge, GRAVY, hydrophobicity and hydrophobic moment of the insertion region) of YJL-4 and several strongly tumor-targeting pHLIP variants (Var0-WT, Var3, and Var7) (Adochite et al. 2014; Demoin et al. 2016) were analyzed using ProtParam and HeliQuest (Table 1). ProtParam analysis revealed that the instability index of YJL-4 was 31.32, indicating that YJL-4 was more stable than pHLIP (Var3) and pHLIP (Var7); additionally, YJL-4 had a GRAVY of -0.092, indicating that YJL-4 has greater polarity than pHLIP (Var0-WT) and pHLIP (Var3). HeliQuest analysis revealed that the hydrophobicity moment of the membrane insertion region of YJL-4 was 0.385, indicating that YJL-4 is more amphiphilic than pHLIP (Var0-WT) and pHLIP (Var7). (2) Analysis of the peptide secondary structure: The secondary structure of the new peptide was analyzed and predicted using PyMOL, HeliQuest, ProtScale, and DNASTAR (Fig. 3).

**Table 1:** Basic parameters of the peptides

Peptides	Sequence	Instability index	Net charge	GRAVY	Hydrophobicity	Hydrophobic moment
YJL-4	ACDDQNPWARYADLLFPTDTLLLDL	31.32	− 4	− 0.092	0.763	0.385
Var0-WT	ACEQNPIYWARYADWLFTTPLLLLDLALLVDADEGT	16.08	− 5	0.3	0.931	0.366
Var3	ACDDQNPWRAYLDLLFPTDTLLLDLLW	41.24	− 4	0.096	0.841	0.389
Var7	ACEEQNPWARYLEWLFPTETLLLEL	50.92	− 4	− 0.2	0.893	0.373

### Synthesis and purification of peptides

YJL-4 and the control peptide kVar7 were successfully synthesized via solid-phase peptide synthesis. HPLC analysis of YJL-4 showed one main peak (96.67%, 13.03 min) and three smaller impurity peaks, and mass spectrometry analysis of the product showed one peak with a m/z value of 3444.59 ([M + H] +) (Additional file 1: Fig. S1). The measured molecular weight (3443.59) of YJL-4 was consistent with the theoretical molecular weight (3442.94).

RP-HPLC analysis of the control peptide kVar7 showed one main peak (97.04%, 12.67 min) and three smaller impurity peaks, and mass spectrometry analysis of the product showed two peaks with m/z values of 3348.12 ([M + H]+) and 3372.03 ([M + Na]+) (Additional file 1: Fig. S1). The measured molecular weights (3347.12, 3349.03) of kVar7 were approximately consistent with the theoretical molecular weight (3347.21).

### CD spectroscopy

**Fig. 4:**
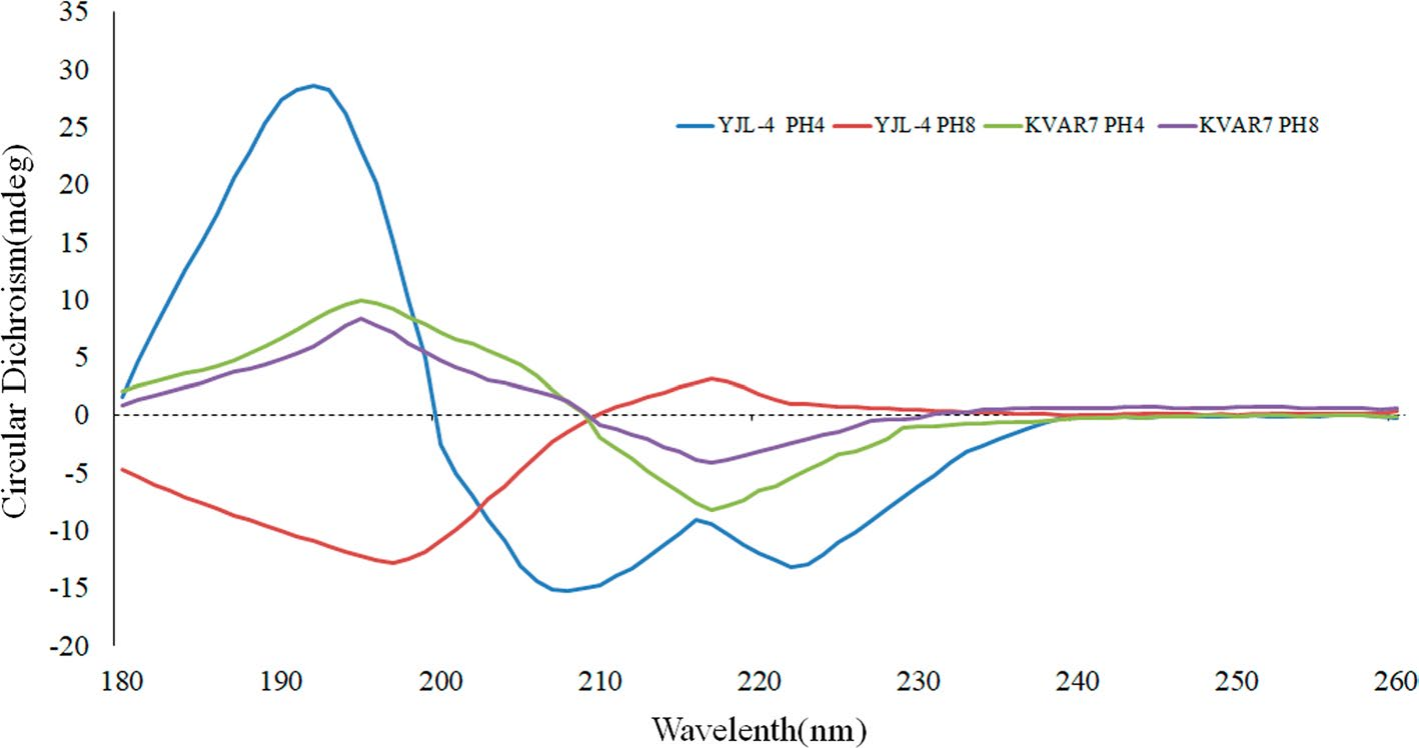
CD spectra of YJL-4 and kVar7 at pH = 4.0 and pH = 8.0

The secondary structures of YJL-4 and the control peptide kVar7 were detected by CD spectroscopy at pH 8.0 and 4.0. In the presence of POPC, YJL-4 exhibited a typical pH-dependent transition from the unstructured conformation to the *α-*helical structure when the pH decreased from 8.0 (red line) to 4.0 (blue line) (Fig. 4). kVar7 was in a β-sheet conformation at pH 8.0 (purple line) and 4.0 (green line) (Fig. 4).

### Radiochemical yield and radiochemical purity of ^68^Ga-YJL-4 and kVar7

The radiochemical yields of ^68^Ga-YJL-4 and ^68^Ga-kVar7 were 89.5 ± 0.16% (n = 3) and 75.8 ± 0.37% (n = 3), respectively. Radio-HPLC analysis of the purified products showed that the purified ^68^Ga-YJL-4 had a radiochemical purity of 97.95 ± 0.06% (n = 3) and a retention time of 10.51 min (Additional file 2: Fig. S2), and kVar7 had a radiochemical purity of 95.3 ± 0.13% (n = 3) and a retention time of 9.58 min.

### Serum stability and serum protein binding

**Fig. 5:**
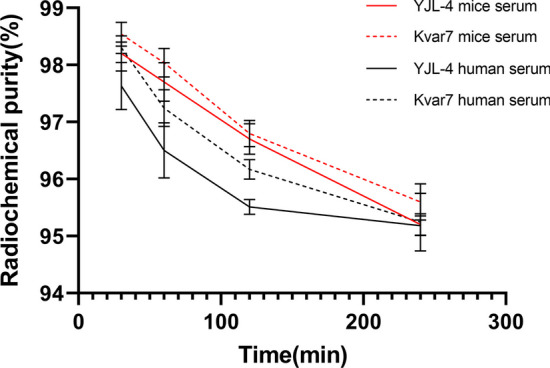
In vitro stability of ^68^Ga-YJL-4 and ^68^Ga-kVar7

^68^Ga-YJL-4 and ^68^Ga-kVar7 were stable, and their radiochemical purities were still as high as 95% after incubation in human serum (or mouse serum) at 37 °C for 240 min (Fig. 5).

**Fig. 6:**
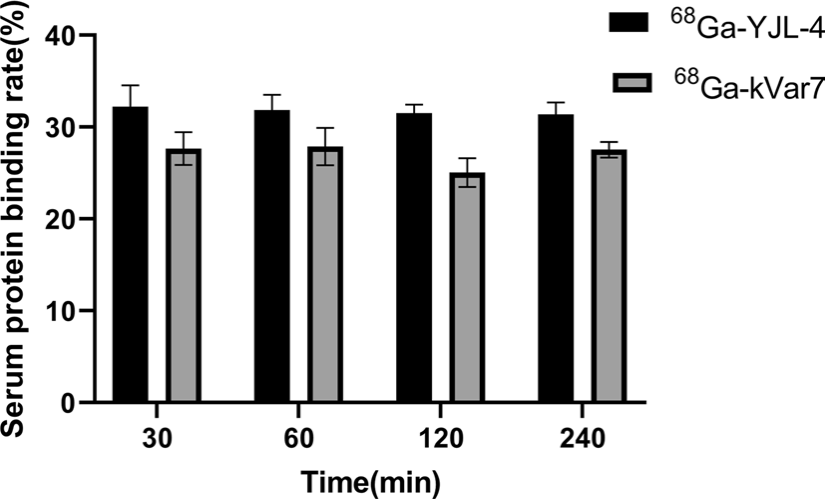
The serum protein binding rates of ^68^Ga-YJL-4 and ^68^Ga-kVar7

The serum protein binding rates of ^68^Ga-YJL-4 and ^68^Ga-kVar7 are shown in Fig. 6. The serum protein binding rates of ^68^Ga-YJL-4 at 30, 60, 120, and 240 min were 32.21 ± 2.35%, 31.83 ± 1.69%, 31.50 ± 0.95%, and 31.39 ± 1.29%, respectively, while those of ^68^Ga-kVar7 were 27.66 ± 1.78%, 27.89 ± 2.02%, 25.04 ± 1.56%, and 27.54 ± 0.86%, respectively.

### In vivo biodistribution

**Fig. 7:**
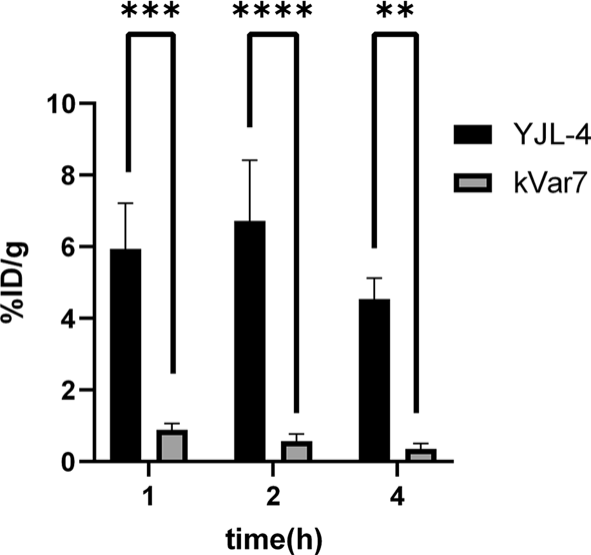
Biodistribution of ^68^Ga-labeled peptides in tumors (n = 3). The distribution of ^68^Ga-YJL-4 in tumors was significantly greater than that of ^68^Ga-kVar7 (**P = 0.0011, ***P = 0.0002, ****P < 0.0001)

**Fig. 8:**
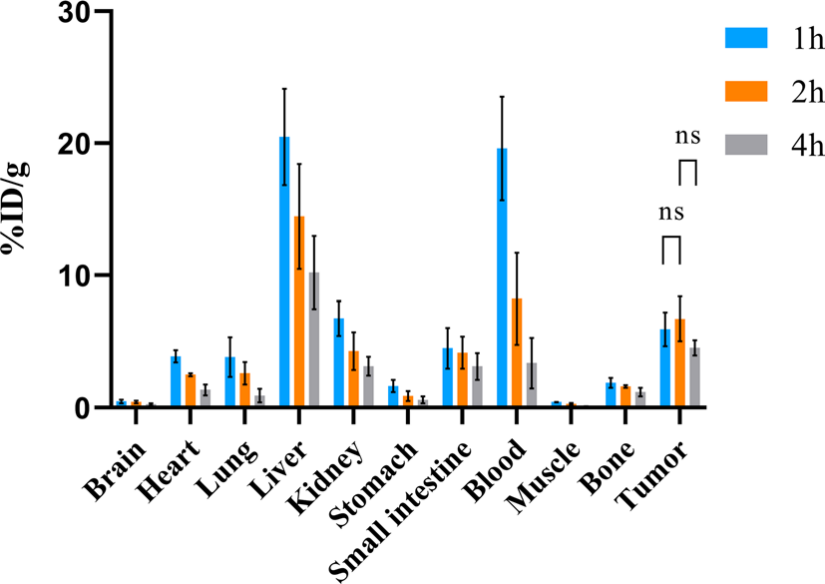
Biodistribution of ^68^Ga-YJL-4 in mice with MDA-MB-231 tumors (n = 3)

**Fig. 9:**
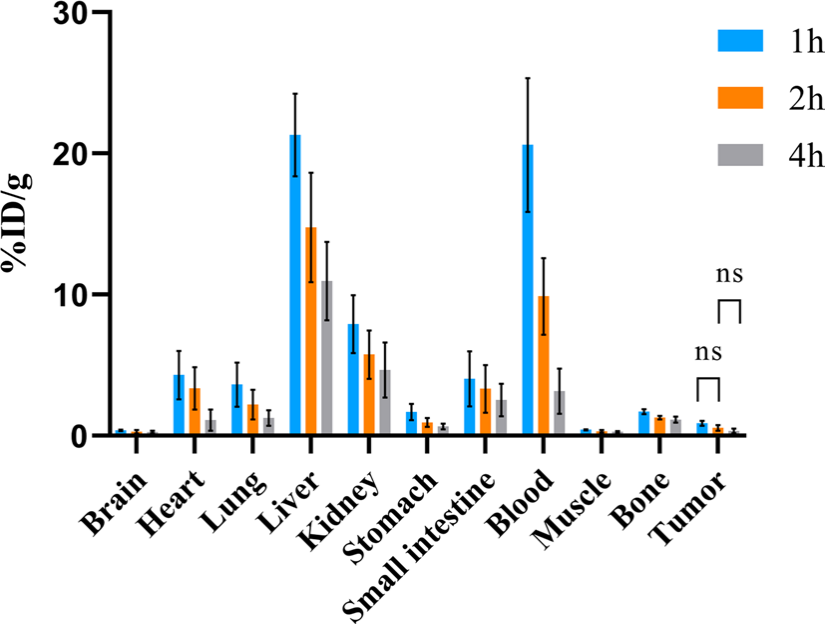
Biodistribution of ^68^Ga-kVar7 in mice with MDA-MB-231 tumors (n = 3)

The biodistribution levels of ^68^Ga-YJL-4 in tumors were 5.94 ± 1.27% ID/g, 6.72 ± 1.69% ID/g and 4.54 ± 0.58% ID/g at 1, 2 and 4 h, respectively, after injection of ^68^Ga-YJL-4 (1.85 MBq/200 μL) into tumor-bearing nude mice via the tail vein, which were higher than the corresponding ^68^Ga-kVar7 biodistribution levels in tumors (*P* < 0.01) (Fig. 7). Among the measured off-target organs, ^68^Ga-YJL-4 and ^68^Ga-kVar7 were highly distributed in the liver and blood (Figs. 8, 9). The distribution of ^68^Ga-YJL-4 in the liver at 1, 2, and 4 h was 20.49 ± 3.64, 14.46 ± 3.98, and 10.22 ± 2.77% ID/g, respectively, and that in the blood was 19.61 ± 3.92, 8.23 ± 3.47, and 3.38 ± 1.91% ID/g, respectively. The distribution of ^68^Ga-kVar7 in the liver at 1, 2, and 4 h was 21.31 ± 2.93, 14.77 ± 3.87, and 10.96 ± 2.77% ID/g, respectively, and that in the blood was 20.60 ± 4.73, 9.88 ± 2.71, and 3.17 ± 1.60% ID/g, respectively.

### Small animal PET imaging

**Fig. 10:**
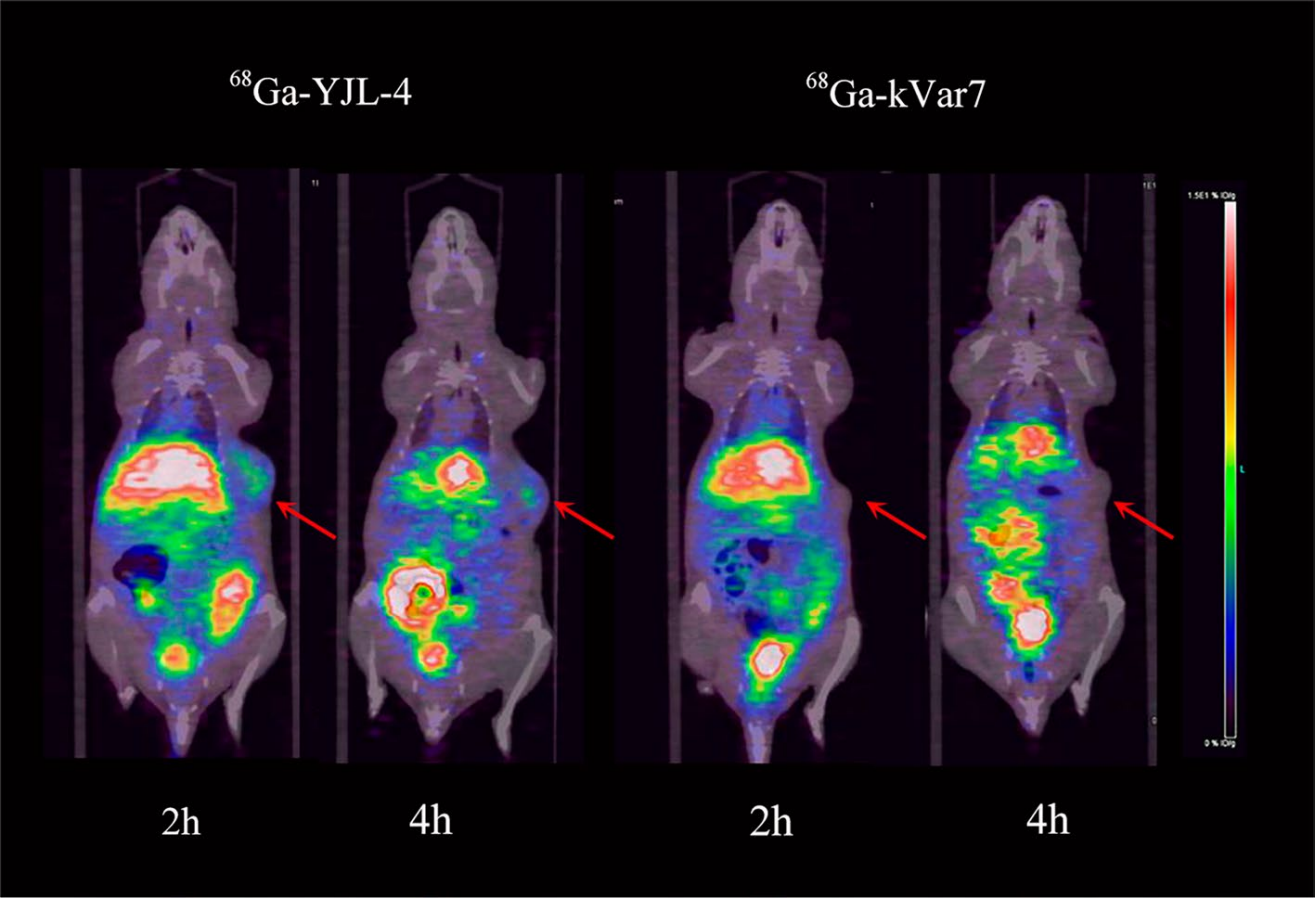
Small animal PET imaging with ^68^Ga-YJL-4 and ^68^Ga-kVar7 in the MDA-MB-231 tumor model

At 2 and 4 h after ^68^Ga-YJL-4 injection, the tumors were clearly visualized by PET, but the tumors in the control group were not visualized. The livers were clearly visualized at each time point after ^68^Ga-YJL-4 or ^68^Ga-kVar7 injection (Fig. 10) (n = 3).

## Discussion

YJL-4 is a novel peptide obtained by selecting the amino acids with the highest frequency in the insertion regions of pHLIPs using a template-assisted method that can design a novel peptide by comparing the sequences of natural peptides and selecting statistically significant residue type (Tossi et al. 2000). Two types of negatively charged amino acids, Asp and Glu, frequently appear in pHLIPs. However, Glu can form hydrogen bonds with the cholesterol contained in normal human cell membranes (Tytler et al. 1995), which is not conducive to the clearance of probes that are not bound to tumor cells. Therefore, Asp was chosen as the negatively charged amino acid in the novel peptide design. ProtParam and HeliQuest analyses of the basic peptide parameters indicated the following: a. The GRAVY of YJL-4 was negative, higher than that of pHLIP(var7) but lower than that of pHLIP(var0) and pHLIP(var3), indicating that the hydrophobicity of YJL-4 was greater than that of pHLIP(var7) and lower than that of pHLIP(var0) and pHLIP(var3), making it relatively easy to synthesize because of its relatively high solubility in aqueous solution compared to that of pHLIP(var0) and pHLIP(var3). b. YJL-4 is a stable peptide (with an instability index of 31.32), and its stability is greater than that of pHLIP(var3) (with an instability index of 41.24) and pHLIP(var7) (with an instability index of 50.92). The high stability enables YJL-4 to remain structurally stable during the labeling process with ^68^Ga. We used ^68^Ga to label NOTA-pHLIP(var7) under the same conditions, but the labeling results were not ideal, which might be related to the relatively poor stability of pHLIP(var7). c. The insertion region of YJL-4 had a hydrophobic moment of 0.385, which was greater than those of pHLIP(var0) (0.366) and pHLIP(var7) (0.373), indicating that the amphiphilicity of the probe was greater than those of pHLIP(var0) and pHLIP(var7). Compared with the peptide pHLIP (Var7) LP that was studied previously by our team (Wu et al. 2022), YJL-4 exhibited improved stability (with an instability index decreasing from 56.99 to 31.32), improved polarity (with a GRAVY decreasing from 0.096 to -0.092) and improved amphiphilicity (with the hydrophobic moment of the cell membrane insertion region increasing from 0.379 to 0.385).

Studies have confirmed that pHLIP family peptides only exhibit an α-helix structure when inserted into the cell membrane in an acidic environment (Reshetnyak et al. 2007, 2008; Gupta et al. 2018). CD analysis of YJL-4 indicated that it could undergo a typical pH-dependent transition from an unstructured conformation to an α-helical structure in an acidic environment, which confirmed that the novel peptide YJL-4 obtained using the template-assisted method still possesses the basic features of pHLIPs and can be inserted into tumor cell membranes in acidic environments. kVar7 is a pH-insensitive peptide (Chen et al. 2022; Sosunov et al. 2013) and is found in a β-sheet conformation at pH 4.0 and 8.0, which means that it will not be inserted into the cell membrane and can thus be used as a negative control.

The biodistribution of ^68^Ga-YJL-4 in tumors was significantly greater than that of ^68^Ga-kVar7 at 1, 2 and 4 h after injection, suggesting that ^68^Ga-YJL-4 has a good ability to target tumors. This high targeting ability may be related to the relatively high amphiphilicity of the probe. The probe was distributed at high levels not only in tumors but also in the liver and blood. The high biodistribution of the probe in the liver might be due to the following reasons: a. the probe is mainly metabolized through the liver pathway, and b. the probe binds strongly to intrahepatic tissue proteins and plasma proteins. The high blood background of the probe might be related to the high binding strength of the probe to serum proteins and the difficult-to-eliminate complex formed.

The PET imaging results were consistent with the biodistribution trends. The tumors were clearly imaged at 2 and 4 h after ^68^Ga-YJL-4 injection, whereas the tumors were not visualized after injection of the control probe, which indicates that the negatively charged Asp residues in ^68^Ga-YJL-4 were neutralized in the acidic TME. Therefore, the peptide changed from a disordered structure to an *α-*helical structure and was inserted into the tumor cell membrane. In contrast, the control probe was still positively charged in an acidic TME and thus was not converted into an *α-*helical structure and inserted into the tumor cell membrane. In addition, both ^68^Ga-YJL-4 and the control probe were found at high concentrations in the mouse liver, which is not conducive to the imaging of abdominal organs; therefore, further improvements, such as changes in the structure of the NOTA chelator, are needed. A study showed that ^68^Ga labeling requires the formation of six covalent bonds to maintain the stability of the labeled compound, which requires that the three carboxyl groups on the NOTA ring cannot be occupied (Ferreira et al. 2012). In a previous study, we used the NOTA chelator (Fig. 2A) for ^68^Ga labeling. Here, one of the carboxyl groups on the NOTA ring was occupied, resulting in very poor image quality, with abnormal liver imaging severely interfering with tumor and abdominal organ imaging (Additional file 3: Fig. S3). In this study, we improved the ability of the NOTA chelator (Fig. 2B) to retain the three carboxyl groups on the NOTA ring. Compared with that in previous studies, the imaging of the tumor was significantly improved at 2 and 4 h after the injection of the probe, and the imaging of the liver was significantly weakened without causing significant interference with the imaging of abdominal organs. In a follow-up study, we will further modify the NOTA chelator to obtain better PET image quality.

## Conclusions

The novel peptide YJL-4 can be converted into an *α-*helical structure that can be easily inserted into the cell membrane in an acidic environment. ^68^Ga-YJL-4 has a high radiochemical yield and good stability and can target TNBC tissue, but it has relatively high biodistribution in the liver and blood; therefore, this probe requires further modification.

### Supplementary Information

**Additional file 1: Fig. S1**. HPLC and MS of YJL-4.

**Additional file 2: Fig. S2**. A representative QC radio-HPLC chromatogram of ^68^Ga-YJL-4.

**Additional file 3: Fig. S3**. Small-animal PET imaging with ^68^Ga-YJL-4 (A 2 h, B 4 h) and that with ^68^Ga-kVar7 (A 2 h, B 4 h).

## Data Availability

The datasets used and/or analyzed during the current study are available from the corresponding author upon reasonable request.

## Abbreviations

CD: Circular dichroism
PET: Positron emission tomography
pHLIP: PH (low) insertion peptide
TME: Tumor microenvironment
TNBC: Triple-negative breast cancer
