# Supplementary figures and images for: Small animal PET imaging with the 68Ga-labeled pH (low) insertion peptide-like peptide YJL-4 in a triple-negative breast cancer mouse model
Source: EJNMMI Radiopharm Chem. 2024 Apr 27;9:33. doi: 10.1186/s41181-024-00267-x (PMC11055835; doi:10.1186/s41181-024-00267-x)

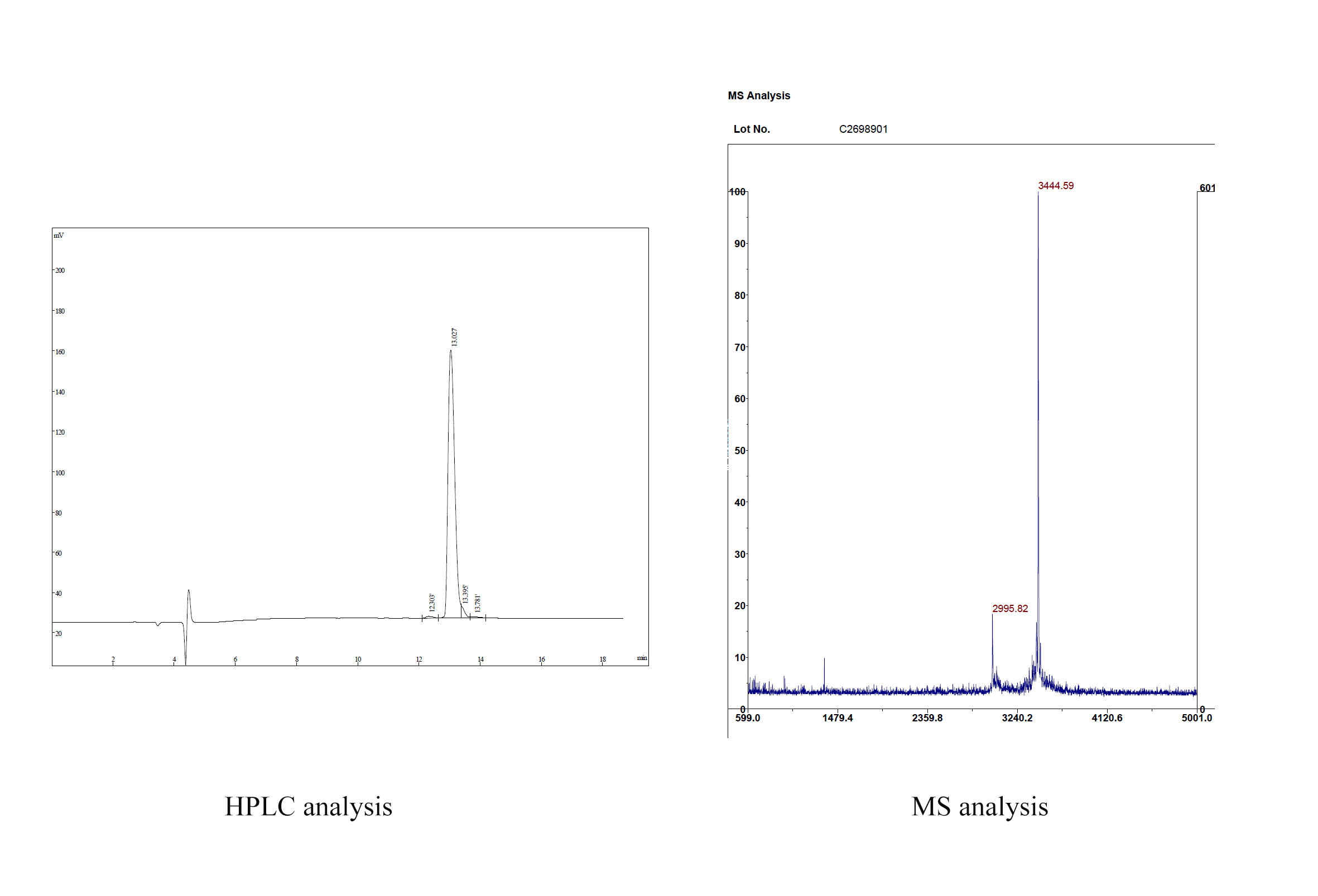

Supplement: Supplementary file 1 — Additional file 1: Fig. S1. HPLC and MS of YJL-4. [file 41181_2024_267_MOESM1_ESM.tif]

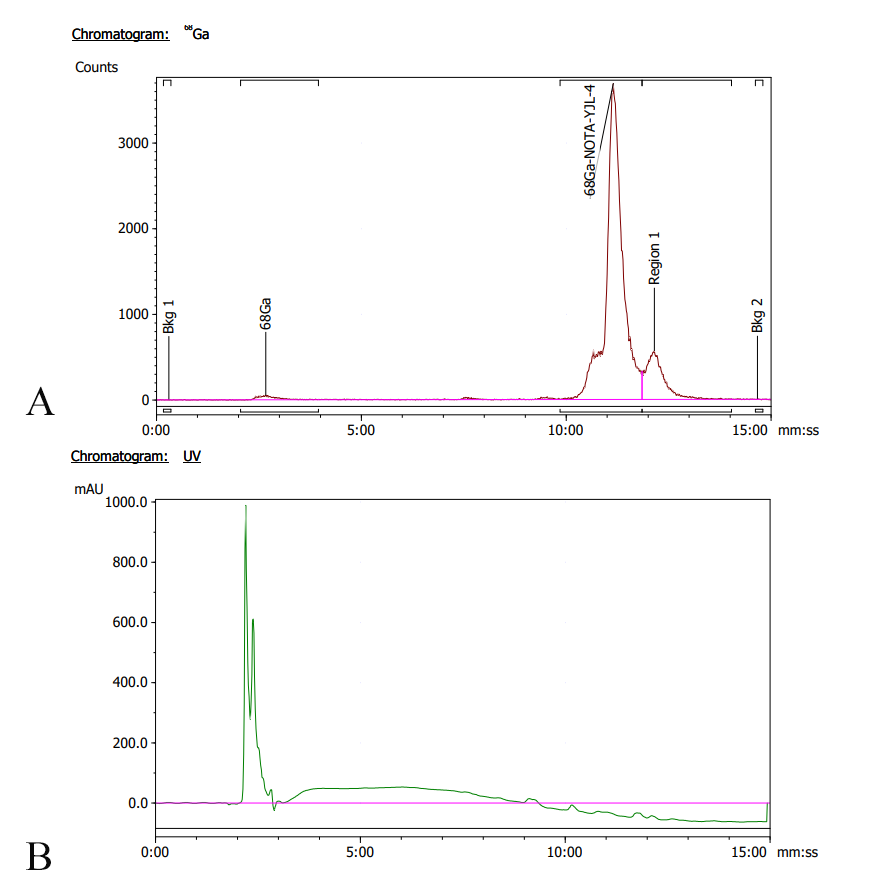

Supplement: Supplementary file 2 — Additional file 2: Fig. S2. A representative QC radio-HPLC chromatogram of 68Ga-YJL-4. [file 41181_2024_267_MOESM2_ESM.tif]

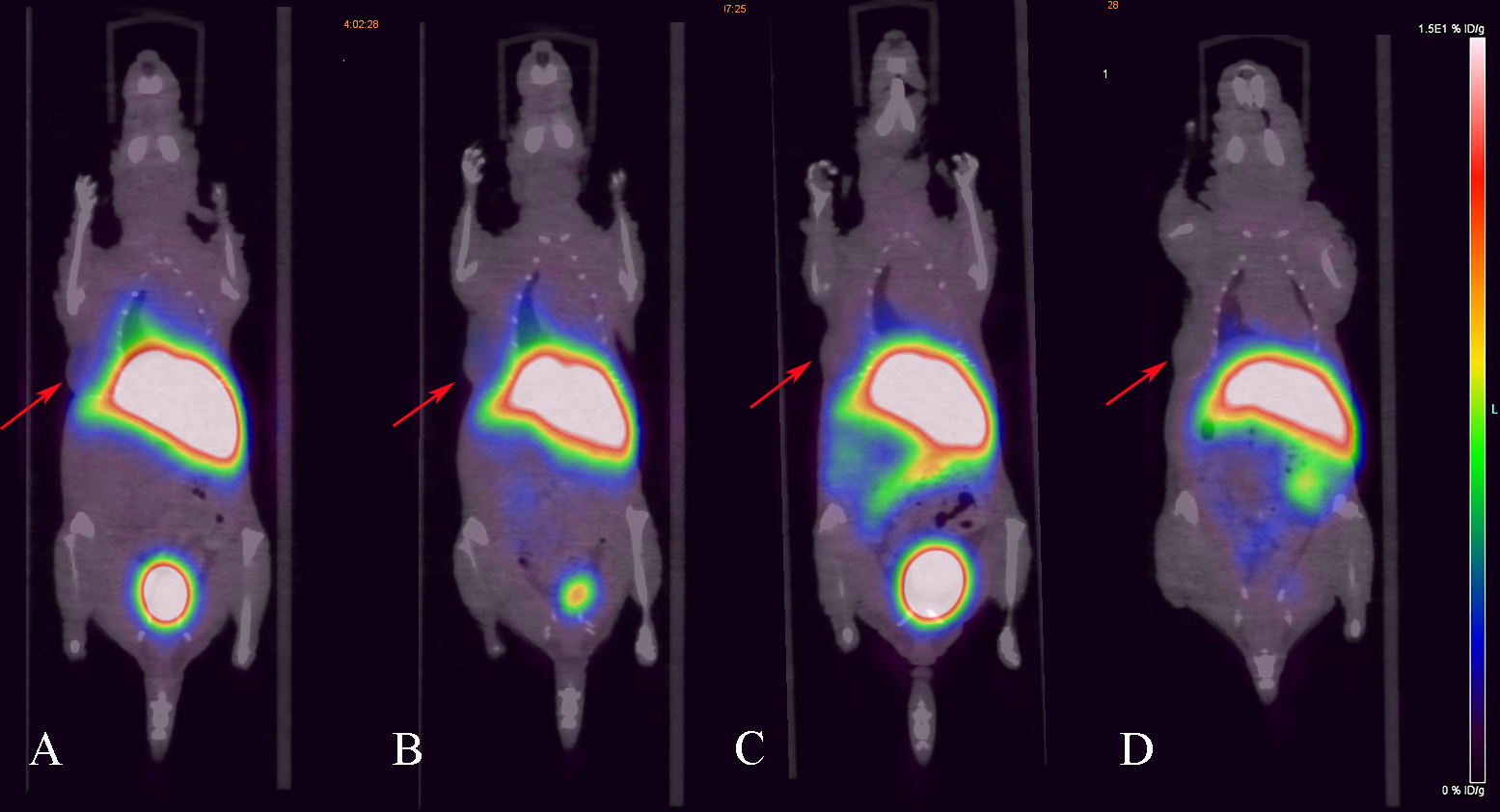

Supplement: Supplementary file 3 — Additional file 3: Fig. S3. Small-animal PET imaging with 68Ga-YJL-4 (A 2 h, B 4 h) and that with 68Ga-kVar7 (A 2 h, B 4 h). [file 41181_2024_267_MOESM3_ESM.tif]
